# The Saving of Time in Dental Operations

**Published:** 1871-01

**Authors:** J. S. Knapp


### ARTICLE VII.

### *The Saving of Time in Dental Operations.*

Read before the American Dental Association, at Nashville.

By Prof. J. S. Knapp, D. D. S.

There is no vocation which demands the observance of the old adage, "Time is Money," more than the practice of Dental Surgery.

There may be those who say they pursue dentistry from the mere love of it, but none will dispute that far the greater number are engaged in it as men engage in other occupations, in order to earn a support for themselves and families. Only those who are fortunate enough either to have gained or inherited a competence, can afford to talk of practicing their profession for the love of it as a fine art.

We should be thorough in what we undertake to do, and slight nothing in the practice of a fine art; but it becomes us to pay especial attention to those plans which are calculated to enable us to perform the greatest good in the shortest time.

If any one present were asked what are the occupations by which the time of dentists is most consumed professionally, he would undoubtedly say that the *construction*, *tempering* and *use of instruments*, the *preparation* and *use of gold*, and *the management of patients.*

First, on the *construction* of *instruments*, I admit that a great variety of these can be found at our dental depots already made; but any dentist who is not able to repoint and shape them in accordance with his wants, is in a *helpless condition* and is *losing time* every day.

Every dentist, besides being qualified with good judgment to select his instruments for *strength*, *durability* and *adaptability* to the purpose for which they are intended, is subjected to great inconvenience in the actual practice of his profession, unless he be able to alter their points, making and tempering them so they can be more readily and effectually used in a manner which he understands better than it is possible for him to know who does not *use* them.

This involves a knowledge of *working* and *tempering steel*, an art which should be pursued in the private laboratory of every practicing dentist, and taught in every dental college.

I have known skillful dentists, and those who were considered to possess profound knowledge in their profession, and who had an extensive practice in a large city, to suffer the greatest inconvenience from the want of this knowledge—the inability to make and temper their own instrument points. The best substitute for the lack of this knowledge would be to be a good draughtsman, in order to explain and show to a manufacturer the exact form of the article required.

So important do I consider this knowledge to insure success that I advise every young practitioner or student, who has not yet been taught the art of working and tempering steel in the laboratory of some dentist or in that of some dental college, to get the information from a practical worker in that metal. When he once knows how to make and temper his own instrument points, he can economize time by preparing them on rainy days, or in hours too early or too late for the usual reception of patients. At any rate the instruments of the dentist should be prepared at such hours as not to interfere with those usually employed in the actual performance of *Dental operations.*

In the use of some of the forms of gold as recommended by certain of our distinguished and able operators, the number of pluggers is large; but in the use of gold in the form of cylinders the necessary number is small, not exceeding ten or twelve for all the pluggers and condensers, thus showing a great saving of time if one uses these instruments and the form of gold for which they are designed.

Excavators, as made by the manufacturers, consist of a variety and number which is too great, and of many shapes which none but a man wanting in judgement would ever attempt to use. This is done by the manufacturer to please the multitude, and probably from the best of motives, but it results in a great loss of time and money. Of course it is well to have as many duplicate points as any individual thinks he will ever have occasion to use; but if ten or twelve excavators be made in accordance with reason, sense and good judgement, an expert operator may properly excavate any cavity to be filled, and in less time, than he who foolishly attempts to use such a great variety of cutting instruments, with each one of which he can accomplish but little. To be plain, no matter what form of gold is to be used, I would discard all the "right and left" (so called) excavators, and many of the forms, five of which are required to make one, and confine myself to the patterns which are but ten or twelve in number. These vary in size and strength, according to the size and location of the cavity in which they are to be used.

Do not deem me dictatorial but allow that I speak from experience, when I say that I should advise all operators to use excavator points in plain, strong, octagonal, ivory handles, with round sockets. These instruments will last any careful man his life time, and are of a size to be handled more effectually than the small steel handles, the points of which are continuous with the handles, making the entire instrument useless in a short time. The points can be untempered and remade when shortened by sharpening, and can be replaced by others when use and repeated pointings have made them too short. I should recommend that the points be bent at different angles, so that some should be but little changed from the line of the handles, others a little more curved, and some bent at a right angle, with the point of connection slightly rounded, and others curved somewhat towards the handle itself, so as to make the "under cut," in cases where it is necessary to form such a cavity.

These excavators are round for half an inch after leaving their sockets, and then gradually squared, to form distinct sides, and flattened in a line right and left of the handle, near the points, so that, besides cutting on their very edges, they will also cut in two ways on each side, making five cutting edges or angles for each excavator point.

Imagine, then, the execution which can be done with one instrument. With six instruments of this kind, almost any cavity that ever was known may be reached, and thoroughly excavated, and prepared for the reception of gold.

When sufficient space has been obtained to properly approach the approximal surfaces of two teeth, both of which are to be filled, two or at most four excavators are all that are necessary to be used; whereas many operators loose time by scraping and cutting away with a dozen in each cavity, and looking and hunting among a confused quantity of these and other instruments to find the one they want?

After using one well formed excavator in one of these cavities, it is a saving of time, without stopping or changing instruments, to turn and use the same cutting point in the opposite place of decay. Such an instrument may cut in the middle and cervical portion of these two cavities, and another, bent at about a right angle with its socket handle, may be made to cut alternately in the opposite half of each cavity, and thus form the "under cutting" in that part which is required. With these two excavators, if properly made and used, the entire orifice of the cavity may also be trimmed, beveled, and smoothed, to aid in giving a fine appearance, finish, and perfection to the plug.

If the cavity to be filled is on the grinding surface of the tooth, with but a small crevice leading to an extensive place of decay, one or two instruments are all that are required to open and enlarge this crevice to a size nearly equal to that of the carious dentine; and two or three excavators are enough, with, perhaps, the aid of one flat head burr and one drill point for the angles, to prepare in a very short time such a cavity for the introduction of the gold.

I am aware that many will tell me that I am talking of executing operations with more simple and fewer instruments than it is practicable to use for such a purpose, and that the rapidity is greater than patients will endure. In regard to the simplicity and small number of instruments which I have represented as capable of such work, any one can prove it by trying them himself, or by seeing others use them.

I have thus spoken of instruments and the rapidity with which they may be used as a general rule, to which, owing to the great variety of cavities, their angles and complications, and the difference in the fortitude of patients and their controllability, there are exceptions.

In the preparation and use of gold there are such widely different views entertained by different operators, that whatever form of this material might be advocated it would be liable to be severely criticised by the advocates and followers of other methods and forms.

I am one of those, who, after having tried gold foil in strips, ropes, and pellets, and sponge or crystal gold (as it is to be used in its invariably slow process of being packed), and gold foil cylinders, have many years since arrived at the conclusion, that in no form can this article be so quickly packed and condensed to a given size and solidity as by the latter method. The cylinders can be prepared before or after office hours, if one is pressed for time, and made in such variety as to be ready to meet the wants of any ordinary case, each one that is introduced being of such a bulk as to fill far more rapidly than it is possible to do with the narrow strip or small particles of crystal gold that are used in the welding process.

If any determined, intelligent and skillful operator will give this form of gold a faithful trial he will like it the more he uses it and will be satisfied that, after its introduction, it is capable of being trimmed and receiving a fine finish in less time than is requisite in fillings of other forms of gold. Practice and perseverance in becoming acquainted with the best methods of making and using cylinders will result in such a saving of time and such facility, that saliva pumps and rubber dams may be discarded, while such solidity is attained that no patient in the hands of a Dental operator need be subjected to the unpleasant jarring operation of malleting, and no dentist need waste his hours in such a tedious and protracted process, with an assistant to aid him in the operation.

In conducting the affairs of a deutal office and the management of patients, there is much to observe by which not merely minutes but hours per day can be saved.

When a Dentist is engaged operating for one who has previously secured the time by appointment, he is liable to many interruptions from a variety of causes. Persons call to make appointments, to pay or collect bills, and some call to have teeth extracted or pain alleviated, and some are in quest of "*cheap dentistry.*" There is no necessity that these hindrances should take up one half the time they usually consume, for the use of a few well chosen words will create promptness in others who would not otherwise feel and see that they are occupying time which belongs to others, and prevent them from taking more of it than is actually necessary.

It may be good policy sometimes to allow a patient undergoing a regular sitting to see an extracting operation, if it be not a protracted and difficult one, but never in the latter case should unconnected parties see what may give them a wrong impression, making them blame the dentist, and perhaps discourage them from undergoing a similar operation.

An operator is excusable for leaving his patient a very short time for all necessary interruptions, but not so for engaging in idle talk with those who have nothing to do but pass their own time in a profitless manner and appropriate that which belongs to another. Neither is he excusable for occupying an unreasonable length of time in any operation.

In extracting teeth, firmness of manner and self-possession combined with gentleness and sympathy will generally operate to inspire confidence, and in most cases prevent that desire on the part of the patient, so often manifested, to *delay*, by which many lose so *much time* and *patience* and numerous *patients*.

We should never deceive children in regard to extracting their teeth, or allow parents to tell them in our presence those falsehoods which so affect them afterwards that they are hard to manage, and serv to make them doubt the truth when they hear it.

In preparing sensitive teeth for filling, it is quite possible for one dentist to manage and operate successfully on a patient for whom another dentist can do nothing. The difference is in deportment, voice and words; and he will save most time who exercises these aright, and who can exert the greatest moral force and will-power over his timid and but half reasoning patient.

I regret that, in this age of progress in dental science, there should still live some who think they will save time by placing arsenic in sensitive teeth, in order that they may afterwards more quickly prepare them for filling.

Perhaps one of the most frequent causes of loss of time to the dentist arises from the ignorance or thoughtlessness of patients. They do not always understand that while talking or rinsing their mouths, they are occupying far more time than is necessary, and that their hour is fast gliding by, and that another patient has a right by appointment to the succeeding hour. It is best to state the facts, and to use a gentle reminder that the time is passing. Better be occupied in actual operations rather than in profitless talk.

Much time may also be saved if the operator will hold the tumbler to the mouth of his patient only as often as he deems it necessary, instead of allowing the patient to handle the glass and rinse *ad libitum*. Young and timid operators frequently lose much time by failing to put their patient in the most convenient position for them to accomplish what is to be done. Time wasted by the patient holding his head over the spittoon may easily be shortened by a gentle touch on the shoulder, when the head goes at once back to its place.

Let us follow these simple directions, and do no cheap dentistry (so called), and make patients pay for the hours they consume, and much more time and money may be saved.—*Dental Register*.

---

